# Circadian Mechanisms: Cardiac Ion Channel Remodeling and Arrhythmias

**DOI:** 10.3389/fphys.2020.611860

**Published:** 2021-01-14

**Authors:** Joyce Bernardi, Kelly A. Aromolaran, Hua Zhu, Ademuyiwa S. Aromolaran

**Affiliations:** ^1^Masonic Medical Research Institute, Utica, NY, United States; ^2^Department of Surgery, The Ohio State University Wexner Medical Center, Columbus, OH, United States

**Keywords:** circadian rhythm, metabolic disorders, autonomic regulation, ion channel remodeling, long QT syndrome, atrial fibrillation

## Abstract

Circadian rhythms are involved in many physiological and pathological processes in different tissues, including the heart. Circadian rhythms play a critical role in adverse cardiac function with implications for heart failure and sudden cardiac death, highlighting a significant contribution of circadian mechanisms to normal sinus rhythm in health and disease. Cardiac arrhythmias are a leading cause of morbidity and mortality in patients with heart failure and likely cause ∼250,000 deaths annually in the United States alone; however, the molecular mechanisms are poorly understood. This suggests the need to improve our current understanding of the underlying molecular mechanisms that increase vulnerability to arrhythmias. Obesity and its associated pathologies, including diabetes, have emerged as dangerous disease conditions that predispose to adverse cardiac electrical remodeling leading to fatal arrhythmias. The increasing epidemic of obesity and diabetes suggests vulnerability to arrhythmias will remain high in patients. An important objective would be to identify novel and unappreciated cellular mechanisms or signaling pathways that modulate obesity and/or diabetes. In this review we discuss circadian rhythms control of metabolic and environmental cues, cardiac ion channels, and mechanisms that predispose to supraventricular and ventricular arrhythmias including hormonal signaling and the autonomic nervous system, and how understanding their functional interplay may help to inform the development and optimization of effective clinical and therapeutic interventions with implications for chronotherapy.

## Introduction

The circadian rhythm is an oscillatory physiological process that occurs within a 24-h period. This rhythmic behavior is evolutionarily conserved and has an critical role in the ability of organisms to modulate endogenous cellular and molecular activities in response to biological cues involving day/night and sleep/wake variations (Andreani et al., 2015). Adverse modulation of circadian rhythms predisposes to sleep disorders and increases risk of cardiovascular diseases and metabolic disorders with significant implications for the quality of life and longevity of patients (Bhupathiraju and Hu, 2016).

The circadian system is composed of a central clock located in the suprachiasmatic nuclei (SCN) of the hypothalamus, consisting of over 20,000 neurons, and peripheral clocks that are present in virtually all tissues. The central clock is synchronized with the environment through external cues, particularly by light, and can entrain peripheral clocks *via* neuronal and humoral factors (Buhr and Takahashi, 2013), such as autonomic tone and glucocorticoid signaling. The rhythm of peripheral clocks can also be regulated by external stimuli, that includes light, food, temperature, physical activity, and sleep. The significance of these distinct regulatory pathways has been extensively discussed in the literature (Xie et al., 2019), and therefore not fully considered in this review. The temporal patterns of food intake have also been identified as a crucial factor that sets the timing (phase) of peripheral clocks (Damiola et al., 2000). Furthermore, the phase of central and peripheral clocks is controlled and/or regulated by different physiological cues, suggesting these phase differences can lead to pathological disease mechanisms that underlie vulnerability to heart failure or cardiovascular diseases.

The molecular machinery of the central and peripheral clocks can be defined by transcriptional/translational feedback loops consisting of the two core transcriptional factors, *CLOCK* and *BMAL1*. These transcription factors have been shown to bind to the enhancer boxes (Ebox) in the promoter region of the negative regulators *PERIOD* (*PER*) and *CRYPTOCHROME* (*CRY*; Gekakis et al., 1998). The PER and CRY proteins accumulate in the cytoplasm, which is then followed by their translocation to the nucleus, where they form a dimer complex which, in turn, suppresses the innate transcriptional activity of *CLOCK* and *BMAL1*, resulting in an oscillatory negative feedback loop mechanism (Buhr and Takahashi, 2013). This core loop is interconnected with a second loop of nuclear receptors, a transcriptional activator ROR (A/B) and a transcriptional repressor REV-ERB (A/B), both of which are activated by the heterodimer CLOCK-BMAL1, that compete for responsive elements in the regulatory sequences of the core clock genes to modulate their transcriptional activities including *BMAL1* (Buhr and Takahashi, 2013). More specifically, ROR (A/B) stimulates *BMAL1* transcription while REV-ERB (A/B) inhibits it (Guillaumond et al., 2005).

**FIGURE 1:**
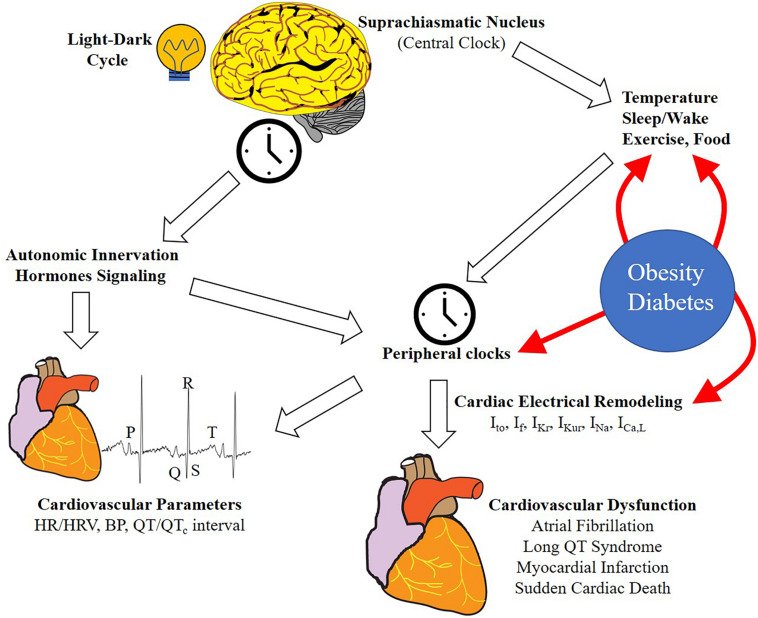
Schematic representation of the regulation of circadian rhythms of cardiovascular function. Light/dark cycle entrains the central clock in the SCN, which in turn regulates rhythmic oscillation in peripheral tissues clocks through neurohumoral signaling. Peripheral clocks are also regulated by other stimuli including sleep/wake, food intake, exercise, and temperature. In the heart, this regulation results in the rhythmicity of different physiological (cardiovascular parameters and ion channel expression) and pathological processes (cardiovascular diseases and arrhythmia). Lifestyle can influence and alter the effect of some external cues (HFD, shiftwork etc.). Metabolic diseases (e.g., obesity and diabetes) can influence the circadian rhythms in different tissues and processes, particularly in the heart, leading to ion channel expression remodeling and increasing the risk of cardiovascular disease (CVD) and arrhythmias. HR, heart rate; HRV, heart rate variability, BP, blood pressure; *I*_*to*_, transient outward potassium current, *I*_*f*_, funny current, *I*_*Kr*_, rapid delayed rectifier potassium current; *I*_*Kur*_, ultra-rapid delayed rectifier potassium current; *I*_*Na*_, sodium current; *I*_*CaL*_, L-type calcium current.

Furthermore, circadian oscillations can also modulate cellular posttranslational processes (Green, 2018), through targeted protein phosphorylation, ubiquitination (Robles et al., 2017), redox and metabolic modulatory pathways (Wang et al., 2012). Approximately 10–40% of the genes expressed in specific tissues follow a circadian pattern and these intrinsic clocks are important for the maintenance of tissue and cellular homeostatic control (Panda et al., 2002; Zhang et al., 2014). A peripheral clock is also known to be present in the heart, where it plays a pivotal role in regulating cardiac electrical excitability, metabolism, and the biophysical properties of major cardiac ionic channels (Bray and Young, 2008; Black et al., 2019). This further highlights a critical role for circadian rhythms in the modulation of cellular mechanisms that contribute to cardiac function in health and disease (Figure 1). In this review we discuss recent studies on circadian rhythms and the pathophysiology of cardiac ion channels. We further discuss the contribution of circadian rhythms in disease states that lead to altered cardiac electrical remodeling with implications for cardiac arrhythmias and cardiovascular disorders in general.

## Circadian Modulation of the Autonomic Nervous System and Ion Channel Regulation

The rhythmic control of cardiac events could be explained by the existence of daily oscillations in several cardiovascular parameters, including heart rate (HR; Furlan et al., 1990), heart rate variability (HRV; Bonnemeier et al., 2003a), blood pressure (BP; Millar-Craig et al., 1978), cardiac output (Cugini et al., 1993) or QT interval duration (Bonnemeier et al., 2003b), and the activity of the autonomic nervous system (ANS). Typically, ANS activity has been indirectly evaluated by measuring HRV, which is affected by HR, an index of sympathovagal balance (Bootsma et al., 1994).

Furthermore, HR, BP, and cardiac output follow diurnal patterns, defined by a morning peak (acrophase) and a nocturnal decrease (nadir) (Degaute et al., 1991; Veerman et al., 1995) and reinforces an important role for these cardiovascular parameters in defining vulnerability to arrhythmogenic events. For example, myocardial infarction, stroke, and ultimately sudden cardiac death (SCD) show a higher prevalence during morning hours.

The slowing of the HR at night, leads to a lengthening of the heart rate corrected QT interval (QT_*c*_) (an index of ventricular repolarization) (Browne et al., 1983). This diurnal variability of repolarization is consistent with the circadian profile of catecholamine circulation (Bexton et al., 1986). In fact, the variations that occur in HR are largely regulated by the two branches of the ANS, the sympathetic and the parasympathetic nervous systems through circulating neurohumoral factors including vasoconstrictive, vasodilative, and proinflammatory cytokines. Nadir in HR diurnal oscillation is generally associated with increased parasympathetic activity at night, while the acrophase is linked with changes in the sympathetic tone during daytime (Furlan et al., 1990).

Despite a critical role for the ANS in circadian rhythms, its contribution to the diurnal variation in HR is not completely clear. The majority of studies suggest that the central clock is not involved in the control of HR by circadian rhythms, as demonstrated in transplanted hearts (Bigger et al., 1996), Langendorff-perfused hearts (Young et al., 2001), cultured cardiomyocyte monolayers (Durgan et al., 2005), possibly due to a lack of an intact autonomic innervation, as well as β-adrenergic receptor deficient-mice (Kim et al., 2008; Swoap et al., 2008) or models of autonomic blockade (Makino et al., 1997; Oosting et al., 1997). Moreover, in pheochromocytoma patients that show sustained and elevated levels of circulating catecholamines, the circadian mediated decrease in BP persists (Statius van Eps et al., 1993), suggesting a role for peripheral clocks in the regulation of these biological parameters. However, Tong et al. (2013) demonstrated that both SCN lesion and pharmacological ANS blockade in mice lead to a loss of circadian rhythmicity in HR, and that ANS seems to influence some cardiac ion channels gene expression.

There are other systemic rhythmic factors, including glucocorticoids (e.g., cortisol) or mineralocorticoids that may also influence the circadian rhythms of the cardiovascular system but with contrasting outcomes associated with diurnal patterns. For example, Shea et al. have demonstrated that the diurnal variation in BP is modulated or controlled by the circadian rhythms in cortisol or catecholamines (Shea et al., 2011). By contrast Imai and others showed that exogenous administration of glucocorticoids changes the rhythmic pattern of BP variations, and prevents the nocturnal-dependent decreases in BP and further suggests an important role for the hypothalamic–pituitary–adrenal axis in influencing the circadian rhythm of BP (Imai et al., 1989).

In native cardiomyocytes, mineralocorticoids, and glucocorticoids have been shown to exert their effects on cellular functions through the mineralocorticoid receptor leading to distinct functional and transcriptional outcomes (Jaisser et al., 2011; Oakley and Cidlowski, 2015). For example, glucocorticoid receptor signaling in cardiomyocytes is critical for the normal development and function of the heart. In contrast, mineralocorticoid receptor signaling in cardiomyocytes participates in the development and progression of cardiac diseases (Imai et al., 1989; Jaisser et al., 2011).

Moreover, there is a paucity of studies that have investigated the potential role of the aldosterone/cortisol-mediated mineralocorticoid receptor in the regulation of the cardiomyocyte circadian clock. However, both Tanaka et al. (2007) and Fletcher et al. (2019), have provided strong evidence for an important link between mineralocorticoid receptor and circadian clock signaling, by demonstrating that aldosterone promotes circadian rhythm dependent functional expression of clock genes (*Bmal1*, *Per1*, *Per2*, and *Rev-ErbA*) in rat cardiomyoblasts and mouse hearts. The β-adrenergic receptor agonist isoproterenol has been shown to increase the circadian rhythms of the *Per2* clock gene in ventricular mice explants (Beesley et al., 2016). This suggests that modulation of the ANS may determine the functional outcomes of cardiac ion channel expression possibly *via* synchronization of the circadian rhythms in the peripheral cardiac clock. It would also be interesting for future circadian rhythm and cardiac studies to evaluate whether mineralocorticoids and glucocorticoids can affect cardiac ion channel expression and promote arrhythmogenesis.

It is widely known that the functional expression of major cardiac ionic channels is critical for normal sinus rhythm and cardiac function. The physiological link between cardiac action potential and its ionic channels is vital for mechanistic insights into the clinical consequences that occur when there are disease-induced changes in the functional properties of these ionic channels.

A critical balance of cardiac ionic depolarizing (Na and Ca channels), and repolarizing mechanisms (K channels), is an important determinant of the duration of the cardiac action potential (AP) and refractory period (Carmeliet, 2006). This means that disease processes that either increase depolarizing currents or decrease repolarizing currents will alter this balance and predispose to reentry and/or induction of ectopic foci, that increases the likelihood of developing arrhythmogenic events (Antzelevitch and Burashnikov, 2011), and ultimately the transition to heart failure and SCD. Research efforts that are directed toward a comprehensive understanding of the link between the cardiomyocyte molecular clock and electrical instability have identified and validated novel mechanistic links associated with oscillatory ion channel expression (summarized in Table 1). Our hope is that these findings will trigger additional research investigations into unappreciated but significant pathways that are directly or indirectly linked to circadian molecular pathways and help to provide insights that will further advance the field of chronological modulation of cardiac function.

**TABLE 1:** Altered functional expression of ion channels by the circadian molecular clocks.

Ion Channel	Channel Subunit	Current	Localization	Circadian Rhythm	Circadian Expression	Assessment Type	Species	References
Na	Na_*v*_1.5	*I*_*Na*_	V	yes	↑ dark	mRNA	rat, mouse	Schroder et al., 2013
	HCN4	*I*_*f*_	SAN	yes	↑ light	mRNA, protein, current	mouse	Wang et al., 2016
Ca	Ca_*v*_1.2	*I*_*Ca,L*_	V	yes	↑ light	protein, current	guinea pig	Chen et al., 2016
K	K_*v*_4.2	*I*_*to*_	A, V	yes	↑ light	mRNA, protein, current	rat, mouse	Yamashita et al., 2003; Jeyaraj et al., 2012
	KChiP2	*I*_*to*_ b subunit	A, V	yes	↑ dark	mRNA, protein	mouse	Jeyaraj et al., 2012
	K_*ir*_3.1/3.4	*I*_*K,ACh*_	A	no		mRNA	rat	Yamashita et al., 2003
	Kv1.5	*I*_*Kur*_	A, V	yes	↑ dark	mRNA, protein, current	rat	Yamashita et al., 2003
	ERG	*I*_*Kr*_	A, V	yes	↑ light	mRNA	mouse, rat	Schroder et al., 2015
	K_*v*_7.1	*I*_*Ks*_	A, V	no		mRNA	mouse, rat	Yamashita et al., 2003; Schroder et al., 2015

*A, atrium, V, ventricle, SAN, sinus atrial node, ↑ dark, increased expression during dark period, ↑ light, increased expression during light period. Modified from Black et al. (2019).*

## Circadian Modulation of Supraventricular and Ventricular Arrhythmias

Supraventricular and ventricular arrhythmias display opposing circadian patterns. Among the supraventricular arrhythmias, atrial fibrillation (AF) is one of the most common arrhythmias in both men and women and it is characterized by increased morbidity and mortality. One major mechanism that underlies the pathogenesis of AF is rapid and disorganized atrial electrical activity that ultimately leads to loss of efficient atrial function, and altered ventricular contraction (Nattel, 2002). This means we need to have a good understanding of how major atrial ionic currents may be modulated in disease states that increase AF risk. Obesity has been shown to be an independent risk factor for AF (Vyas and Lambiase, 2019; Javed et al., 2020), suggesting that understanding how obesity-related mechanisms modulate ion channel function may inform effective pharmacological and dietary interventions in patients.

Electrical remodeling in AF includes increases in the pacemaker current, *I*_*f*_ (Lai et al., 1999), a strong reduction of the transient outward (*I*_*to*_) and the ultra-rapid (*I*_*Kur*_) K current densities (Brandt et al., 2000), and a significant reduction in the L-type Ca current, *I_*Ca*,*L*_* (Christ et al., 2004), which is one of the most consistent features. Furthermore, constitutive activation of the acetylcholine-activated K current (*I_*K*,*Ach*_*), is important for the maintenance of chronic AF (Dobrev et al., 2005). AF incidence is higher during nighttime, and this has been confirmed in ICD data reported by Shusterman et al. (2012). This nocturnal prevalence is consistent with a predominance of vagal activity at night that can stimulate *I*_*K,ACh*_ in atrial cardiomyocytes and inhibits *I*_*f*_ and *I*_*Ca,L*_, thus promoting a shortening of refractory period and reentry (Chen et al., 2014). There are also more recent reports of the contribution of altered function of the rapidly (*I*_*Kr*_) and slowly (*I*_*Ks*_) activating components of the delayed rectifier K currents (*I*_*K*_) in AF (Caballero et al., 2010; Gonzalez de la Fuente et al., 2013). We have recently shown increased current density of the delayed rectifier K current (composed of *I*_*Kr*_ and *I*_*Ks*_) in a high-fat diet (HFD)-induced obese guinea pig model (Martinez-Mateu et al., 2019), with implications for an abbreviated atrial AP duration (APD), and propensity for AF tachycardia (Martinez-Mateu et al., 2019). There is a paucity of arrhythmia studies that investigate the modulation, by the cardiomyocyte molecular clock, of *I*_*K*_ function in metabolic disorders. The important role of delayed rectifier K currents in limiting cardiac repolarization in health and disease suggests that future studies that investigate their modulation by the cardiomyocyte molecular clock are likely to reveal crucial mechanistic insights that will inform targeted interventions with implications for precision medicine. Alterations in tissue properties (or impaired tissue structural integrity), and autonomic (manifested as altered sympathovagal activity) remodeling (Nattel, 2002), also predispose to AF risk.

Circadian rhythms in HR is widely attributed to variations in sympathovagal tone (Bootsma et al., 1994). Recent reports have provided evidence that HR diurnal oscillations could also be due to intrinsic circadian rhythms in the activity of the pacemaker of the heart or the sinus atrial node (SAN; Wang et al., 2016). The hyperpolarization activated cyclic nucleotide gated K channel (HCN)4 currents have been proposed to contribute to several functions including pacemaker activity in heart and brain, control of resting membrane potential, and neuronal plasticity (DiFrancesco and DiFrancesco, 2015). The hyperpolarization-activated “funny” current (or *I*_*f*_), is carried by HCN channels, which exists in native cells as heterotetramers built of four HCN subunits (Novella Romanelli et al., 2016). The transcript and protein expression of HCN4 in mice SAN biopsies have been shown to exhibit circadian rhythm profiles compatible with the oscillations of HR. The density of *I*_*f*_ was double at the start of the awake period (higher HR) compared to the sleep period (lower HR) (Wang et al., 2016). Moreover, an *in silico* analysis of the *Hcn4* promoter has revealed the presence of conserved Ebox binding sites for the Clock-Bmal1 heterodimer (Wang et al., 2016), suggesting that its expression may be directly under the control of the cardiomyocyte molecular clock.

Gene transcripts, protein expression, and current densities of the *I*_*Kur*_ channel subunit K_*v*_1.5 and *I*_*to*_ subunit K_*v*_4.2 have shown significant circadian variations in rats. K_*v*_1.5 is increased during the dark period, while K_*v*_4.2 displayed a completely reverse pattern, with an increase during the light period (Yamashita et al., 2003). Moreover, the reversal of light stimulation for 2-weeks attenuated and reversed the circadian pattern of these channel transcripts, while β-adrenergic stimulation solely influenced oscillation in K_*v*_1.5, suggesting that rhythmicity of both channels could be the result of multiple factors (internal cardiomyocyte clock, light/dark cycle, ANS activity, etc.) (Yamashita et al., 2003).

In contrast to AF, ventricular arrhythmias, including ventricular tachycardia (VT) and ventricular fibrillation (VF), are prevalent during morning hours (Siegel et al., 1992; Englund et al., 1999). One potential mechanism is possibly through increases in sympathetic activity after awakening, with β-adrenergic stimulation promoting Ca overload, afterdepolarizations, and reentry mechanisms, and therefore acting as substrates for pro-arrhythmic triggers (Gardner et al., 2016).

These observations emphasize a role for the involvement of sympathetic stimulatory pathways in the propensity and prevalence of SCD in the mornings and reinforces the importance of targeted clinical interventions that utilize β-blockers to limit the morning peaks in SCD especially after myocardial infarction (Peters et al., 1989). Furthermore, circadian variation studies (based on 24-h ECG monitoring), have also been described for distinct ventricular arrhythmias. For example, long QT Syndrome Type 1 (LQT1) and long QT Syndrome Type 2 (LQT2), display a morning prevalence, while LQT Type 3 and Brugada Syndrome, have been shown to display increased incidence at night (Stramba-Badiale et al., 2000; van den Berg et al., 2006; Takigawa et al., 2012).

Long QT Syndrome Type 2 is caused by mutations in the *KCNH2* gene leading to a loss of function of the K_*v*_11.1 (hERG) channel, and pathological decreases in the repolarizing *I*_*Kr*_ current (Curran et al., 1995; Puckerin et al., 2016). Two different variants of the ERG subunit, ERG 1a and ERG 1b, are expressed in human ventricle (Jones et al., 2004) and functional *I*_*Kr*_ is likely to consist of a combination of both variants (hERG 1a/1b) (London et al., 1997). Interestingly, compared with homomeric hERG 1a currents, hERG 1a/1b currents exhibit a twofold increase in density, rate of activation, recovery from inactivation, and deactivation (Sale et al., 2008; Aromolaran et al., 2016; Puckerin et al., 2016; Martinez-Mateu et al., 2019). It has been demonstrated that reducing hERG 1b subunit levels alters *I*_*Kr*_ kinetics and leads to cellular manifestations of pro-arrhythmia, such as APD prolongation and early afterdepolarizations (EADs), in human induced pluripotent stem cell-derived ventricular cardiomyocytes (hiPSC-CMs; Jones et al., 2014). The expression of hERG channels have been reported to follow a circadian variation, and its diurnal pattern is disrupted after cardiac-specific *Bmal1* knockout, suggesting that its control is under the cardiomyocyte molecular clock (Schroder et al., 2015). Compatible with a decrease in gene expression, *I*_*Kr*_ density in the *Bmal1* cardiac knockout was 50% smaller than in control ventricular myocytes, with no differences in gating properties (Schroder et al., 2015). In this study, the specific contribution of the distinct hERG variants to this outcome was not examined. Thus, it would be of particular interest to evaluate if the subunits are under differential circadian control, particularly considering the differences in biophysical properties of channel function, and the implication in a variety of cardiovascular disease conditions.

In a recent retrospective study in heart failure patients, an increase in QT and QT_*c*_ diurnality (QT_*d*_ and QT_*c,d*_), representing the amplitude of their diurnal variation, has been associated with ventricular arrhythmias (Du Pre et al., 2017). The QT_*d*_ and QT_*c*,*d*_ have also been shown to be increased in both congenital (LQT2) or drug-induced (Sotalol) ERG channel dysfunction (Du Pre et al., 2017), supporting the hypothesis that loss of circadian control of ion channel functional expression leads to adverse cardiovascular parameters and increased incidence of arrhythmias.

In human ventricular myocytes *I*_*Kr*_ and *I*_*Ks*_, together with *I_*Ca*,*L*_*, are important determinants of APD. This dynamic ion channel relationship underscores the relevance of cardiac repolarization reserve, which would be expected to limit vulnerability to arrhythmia risk by maintaining normal cardiac repolarization (Carmeliet, 2006). A novel clock-dependent oscillator, Kruppel-like factor 15 (Klf15) has been identified as a rhythmic regulator of repolarization. It has been shown to target the rhythmic expression of the α-subunit (K_*v*_4.2) and the regulatory β-subunit (KChiP2), of the *I*_*to*_ current (Jeyaraj et al., 2012). Both *Klf15* deletion and overexpression in mice led to modification of *I*_*to*_ density and APD with corresponding alterations in the QT interval length, resulting in increased susceptibility to arrhythmias. This is supported by the evidence that an ECG pattern (ST-segment changes), similar to that found in Brugada syndrome, has been observed after deletion of *Klf15* in mice (Jeyaraj et al., 2012).

Expression levels of several other K channels without a circadian pattern were lower in *Bmal1* mice knockout hearts compared to control, suggesting that cardiomyocyte clock signaling might indirectly contribute to the expression of non-circadian K^+^ channels genes (Schroder et al., 2015). Furthermore, in the *Bmal1* mice model, the authors demonstrated a loss of rhythmic expression of SCN5A, which encodes for the cardiac voltage-gated Na channel, with a reduction of the corresponding current *I*_*Na*_ (50%), a slowed HR and an increased incidence of arrhythmias in mice and rat ventricular myocytes (Schroder et al., 2013). It would be of particular interest to evaluate whether oscillations in Na channels are altered in LQT3 patients.

In guinea pig ventricular myocytes, Clock-Bmal1 heterodimers have been shown to regulate the circadian expression and function of L-type Ca channels, and this occurs through the PI3K-Akt signaling pathway, with corresponding oscillations in APD (Chen et al., 2016). We and others have shown that *I*_*Ks*_ and *I*_*Kr*_ contribute prominently to cardiac repolarization in guinea pig ventricular myocytes (Sanguinetti and Jurkiewicz, 1990; Bryant et al., 1998; Aromolaran et al., 2014, 2018). To our knowledge, there have been no reports of diurnal variations in *I*_*Ks*_ and *I*_*Kr*_ functional expression in guinea pig ventricular myocytes. Pathological decreases in *I*_*Ks*_ either due to congenital or inherited mutations in KCNQ1 channel subunits (Aromolaran et al., 2014; Puckerin et al., 2016), or acquired in disease states delay cardiac repolarization leading to prolongation of the QT interval (or LQT1), a disease mechanism that increases vulnerability to fatal arrhythmias such as *Torsades des Pointes* (Khan, 2002; El-Sherif and Turitto, 2003). Therefore, it is important to determine whether these ion channels may be regulated by circadian regulation. This premise is underscored by a previous report by Schroder and others (Schroder et al., 2015) showing that the molecular clock in the heart regulates the circadian expression of KCNH2 (which encodes the hERG channel) and modifies channel gene expression. The authors suggested that a disruption of cardiomyocyte circadian clock mechanisms likely unmasks the diurnal changes in ventricular action potential repolarization and predispose to an increased risk of fatal arrhythmias that underlie SCD. It will be important to determine whether similar mechanisms control cardiac KCNQ1-*I*_*Ks*_ channel functional expression.

Together, it is intriguing to speculate that modulation by circadian rhythms of ion channel functional expression and ANS activity may underlie alterations in the day/night pattern of arrhythmias and SCD.

## Time Restricted Feeding, Metabolic Disorders, Ion Channel Biophysics and Circadian Rhythm Pathways

Changes in the intracellular concentration of several metabolites (e.g., heme, NAD/NADH, CO, glucose, AMP/ATP, etc.) can influence the activity of the clock machinery by regulating histone modifications, DNA interactions or protein modifications (Panda, 2016). Extracellular factors, including hormones and temperature that regulate the peripheral clocks permit their alignment with the central clock. These rhythmic patterns enable a temporal separation of distinct biochemical pathways in a more energy-efficient fashion (Panda, 2016), such that misalignment of central and peripheral clock phases may promote the development of metabolic diseases. Additionally, dietary habits associated with excessive feeding can affect circadian rhythms in distinct organs leading to a higher likelihood of developing the metabolic syndrome (Pickel and Sung, 2020; Figure 1).

Daily eating patterns (feeding-fasting cycle and day *vs* night meals), and time-restricted feeding (TRF) can affect peripheral circadian rhythms. For example, experiments conducted in mice fed *ad libitum* or exposed to TRF have shown how quantity, quality and timing of food intake can alter circadian rhythm physiology. Mice exposed to HFD *ad libitum* (used to induce obesity) have altered diurnal oscillations in hepatic transcriptome, compared to mice fed a standard diet (Eckel-Mahan et al., 2013). Moreover, TRF of HFD improves molecular oscillations (similar to mice fed a standard diet) (Hatori et al., 2012), and therefore suggests its potential ability to attenuate the adverse metabolic consequences of diet-induced pathologies. This suggestion is further reinforced by the demonstration that TRF is able to reduce age-dependent or HFD-dependent deterioration of cardiac function in insects (Gill et al., 2015), and that implementation of a 10-hour TRF may promote weight loss and improve sleep in humans (Gill and Panda, 2015). Moreover, changes in metabolism, as seen after TRF, can lead to an uncoupling of peripheral oscillators from the central clock, with consequent alterations of the phase of circadian gene expression in different tissues, including the heart, while not affecting the SCN clock genes (Damiola et al., 2000).

Obesity and diabetes are functionally related to alterations in circadian rhythms with an impact on cardiac function. Studies on the effect of obesity on circadian rhythmicity of cardiometabolic functions are limited, but obesity has been associated with a decrease in HRV and with a shift in its circadian pattern (Rodriguez-Colon et al., 2014). Notably, polymorphisms in the *CLOCK* gene have been associated with a differential incidence of obesity in humans, further supporting the idea that circadian rhythms have a pivotal role in the development of metabolic syndrome (Scott et al., 2008).

Diabetes leads to alterations in circadian rhythms and adversely affects cardiac function. This functional remodeling process is exemplified by circadian rhythm studies in a rat model of streptozotocin-induced diabetes (Young et al., 2002). The authors demonstrated that diabetes-induced alteration of circulating humoral factors, leads to a loss of normal synchronization of the peripheral heart clock (Young et al., 2002). This observation is further supported by pathological diurnal variations in diabetes biomarkers (including insulin, leptin, glucocorticoids, growth hormone, glucose, and circulating of free-fatty acids) (Ortiz-Caro et al., 1984; Velasco et al., 1988; Havel et al., 1998), and ANS activity (Bernardi et al., 1992). Moreover, two different *BMAL1* SNP haplotypes have been shown to be associated with type 2 diabetes and hypertension in patients, suggesting an important contribution of *BMAL1* variants to the pathogenesis of these disease mechanisms (Woon et al., 2007).

The circadian rhythm distribution of the onset of cardiovascular events is also altered in diabetes. For example, compared to non-diabetic patients the peak in acute myocardial infarction is lower in the morning, and this is followed by a second peak in the evening, with the risk of developing myocardial infarction higher during the nighttime (Hjalmarson et al., 1989). This chronological-dependent susceptibility to myocardial infarction can be explained by alterations in the circadian patterns associated with sympathovagal balance in diabetic patients that display a range of autonomic abnormalities (Bernardi et al., 1992).

There is also evidence of lower parasympathetic activity during the night, and a marked dominance in sympathetic tone in diabetic patients during both day and night (Bernardi et al., 1992). Furthermore, diabetic patients, particularly those with autonomic neuropathy, showed no decrease in BP during the night when compared to non-diabetic patients. This disruption of the circadian rhythm dependent modulation of BP is frequently associated with a poor prognosis (Felici et al., 1991). Therefore, prolonged sympathetic activity in diabetic patients may counteract the protective effect of parasympathetic tone on the cardiovascular system, which normally would then be manifested by a lower incidence of cardiac events during the nighttime (Bernardi et al., 1992). Diurnal differences in the ECG have been observed during hypoglycemia and this is generally manifested as a larger prolongation in QT_*c*_ interval throughout the daytime, suggesting a higher vulnerability to arrhythmias; while the incidence of bradycardia episodes was found to be increased during the sleep cycle (Andersen et al., 2020).

## Future Directions and Conclusions

There is increasing evidence that cardiac diseases can be influenced by circadian rhythms, and peripheral clocks can be altered in the setting of different pathologies, including diabetes, obesity, and hypertension (Maury et al., 2010). There is a lack of progress in the knowledge of arrhythmia mechanisms. However, in recent years there has been a great deal of effort to understand the molecular mechanisms of circadian rhythms that regulate cellular mechanisms in health and disease. For example, hiPSC-CMs have been widely used as disease models for arrhythmias (including LQT) (van Mil et al., 2018) and have been validated as reliable sources for drug safety studies and the assessment of a new drugs pro-arrhythmic risk with translational implications in patients. The evidence that differentiated hiPSCs acquire and exhibit circadian variation in clock genes (Umemura et al., 2019; Kaneko et al., 2020), provides the rationale for the use of these cells in circadian rhythms studies that could provide relevant mechanism-based insights that may be better predictive of disease penetrance with significant implications in patients.

The existence of circadian variations in the manifestation of cardiac events and arrhythmogenesis highlights the critical link between chronotherapy and cardiovascular disorders, particularly arrhythmias. This suggests that the timing of dietary or therapeutic interventions may be key to limiting the incidence of disease mechanisms that impact the quality of life of patients. Several clinical trials have demonstrated a better tolerability and increased efficacy for chronotherapy compared to non-time-based treatment for different pathologies (Levi et al., 1985; Giacchetti et al., 2006; Buttgereit et al., 2008), while some other trials have failed to establish a similar and positive outcome (Levi and Okyar, 2011). This could be attributed to inter-individual circadian differences that can result from sex, age, lifestyle, genetic or disease profile. Therefore, a further understanding of the mechanisms involved in circadian regulation of biological processes is required to further improve the rigor of these approaches.

Existing molecular mechanisms of how circadian rhythms may modulate cardiovascular function are obtained in rodent models including mice and rats, that unlike humans, are nocturnal. Therefore, future studies that incorporate mechanisms in larger animal models are more likely to be rewarded with additional and/or novel mechanism-based insights that could be better translated into therapeutic and clinical significance.

There is also the added complexity of species differences associated with rational development of targeted therapeutics in patients with cardiovascular diseases. This is also because most of the current knowledge about the regulation of genes (ion channels and metabolic factors) targeted by the molecular clock, have been obtained in animal models of clock component manipulation, mainly *Bmal1* and *Clock*. Therefore, we need to exercise caution in the interpretation of outcomes in future studies due to an indirect effect of clock gene modulation, in models where these modulations may not be tissue specific. Therefore, a further analysis of clock genes and associated upstream and downstream molecular pathways could inform or potentially shift current paradigm of the circadian rhythms-dependent regulation of the cardiovascular system, and more specifically arrhythmia substrates that promote ion channel dysfunction.

## Author Contributions

JB and AA researched the concepts. JB wrote the first draft of the manuscript. HZ and KA edited and finalized the manuscript. AA obtained funding, conceived of, wrote, and finalized the manuscript. All authors contributed to the article and approved the submitted version.

## Conflict of Interest

The authors declare that the research was conducted in the absence of any commercial or financial relationships that could be construed as a potential conflict of interest.

## Abbreviations

AF: atrial fibrillation
ANS: autonomic nervous system
AP: action potential
APD: action potential duration
BP: blood pressure
CVD: cardiovascular disease
EAD: early afterdepolarization
ECG: electrocardiogram
ERG: ether-A-go-go-related gene
HR: heart rate
hiPSC-CM: human induced pluripotent stem cell-derived cardiomyocyte
*I*_*Ca,L*_: L-type calcium current
*I*_*f*_: funny current
*I*_*K,ACh*_: acetylcholine-activated potassium current
*I*_*Kr*_: rapid delayed rectifier potassium current
*I*_*Ks*_: slow delayed rectifier potassium current
*I*_*Kur*_: ultra-rapid delayed rectifier potassium current
*I*_*Na*_: sodium current
*I*_*to*_: transient outward potassium current
LQTS: long QT syndrome
SAN: sinus atrial node
SERCA: sarcoplasmic reticulum Ca^2+^-ATPase
SCD: sudden cardiac death
SCN: suprachiasmatic nuclei
TRF: time-restricted feeding
VF: ventricular fibrillation
VT: ventricular tachycardia.

## References

[B1] Andersen A. Jørgensen P. G. Knop F. K. Vilsbøll T. (2020). Hypoglycaemia and cardiac arrhythmias in diabetes. *Ther. Adv. Endocrinol. Metab.* 11:2042018820911803. 10.1177/2042018820911803 PMC7238305 32489579

[B2] Andreani T. S. Itoh T. Q. Yildirim E. Hwangbo D. S. Allada R. (2015). Genetics of circadian rhythms. *Sleep Med. Clin.* 10 413–421. 26568119 10.1016/j.jsmc.2015.08.007 PMC4758938

[B3] Antzelevitch C. Burashnikov A. (2011). Overview of basic mechanisms of cardiac arrhythmia. *Card. Electrophysiol. Clin.* 3 23–45. 10.1016/j.ccep.2010.10.012 21892379 PMC3164530

[B4] Aromolaran A. S. Colecraft H. M. Boutjdir M. (2016). High-fat diet-dependent modulation of the delayed rectifier K(+) current in adult guinea pig atrial myocytes. *Biochem. Biophys. Res. Commun.* 474 554–559. 10.1016/j.bbrc.2016.04.113 27130822

[B5] Aromolaran A. S. Srivastava U. Alí A. Chahine M. Lazaro D. El-Sherif N. (2018). Interleukin-6 inhibition of hERG underlies risk for acquired long QT in cardiac and systemic inflammation. *PLoS One* 13:e0208321. 10.1371/journal.pone.0208321 30521586 PMC6283635

[B6] Aromolaran A. S. Subramanyam P. Chang D. D. Kobertz W. R. Colecraft H. M. (2014). LQT1 mutations in KCNQ1 C-terminus assembly domain suppress IKs using different mechanisms. *Cardiovasc. Res.* 104 501–511. 10.1093/cvr/cvu231 25344363 PMC4296111

[B7] Beesley S. Noguchi T. Welsh D. K. (2016). Cardiomyocyte circadian oscillations are cell-autonomous, amplified by beta-adrenergic signaling, and synchronized in cardiac ventricle tissue. *PLoS One* 11:e0159618. 10.1371/journal.pone.0159618 27459195 PMC4961434

[B8] Bernardi L. Ricordi L. Lazzari P. Soldá P. Calciati A. Ferrari M. R. (1992). Impaired circadian modulation of sympathovagal activity in diabetes. a possible explanation for altered temporal onset of cardiovascular disease. *Circulation* 86 1443–1452. 10.1161/01.cir.86.5.1443 1423958

[B9] Bexton R. S. Vallin H. O. Camm A. J. (1986). Diurnal variation of the QT interval–influence of the autonomic nervous system. *Br. Heart J.* 55 253–258. 10.1136/hrt.55.3.253 3513807 PMC1232162

[B10] Bhupathiraju S. N. Hu F. B. (2016). Epidemiology of obesity and diabetes and their cardiovascular complications. *Circ. Res.* 118 1723–1735. 10.1161/circresaha.115.306825 27230638 PMC4887150

[B11] Bigger J. T. Jr Steinman R. C. Rolnitzky L. M. Fleiss J. L. Albrecht P. Cohen R. J. (1996). Power law behavior of RR-interval variability in healthy middle-aged persons, patients with recent acute myocardial infarction, and patients with heart transplants. *Circulation* 93 2142–2151. 10.1161/01.cir.93.12.2142 8925583

[B12] Black N. D’Souza A. Wang Y. Piggins H. Dobrzynski H. Morris G. (2019). Circadian rhythm of cardiac electrophysiology, arrhythmogenesis, and the underlying mechanisms. *Heart Rhythm* 16 298–307. 10.1016/j.hrthm.2018.08.026 30170229 PMC6520649

[B13] Bonnemeier H. Richardt G. Potratz J. Wiegand U. K. Brandes A. Kluge N. (2003a). Circadian profile of cardiac autonomic nervous modulation in healthy subjects: differing effects of aging and gender on heart rate variability. *J. Cardiovasc. Electrophysiol.* 14 791–799. 10.1046/j.1540-8167.2003.03078.x 12890036

[B14] Bonnemeier H. Wiegand U. K. Braasch W. Brandes A. Richardt G. Potratz J. (2003b). Circadian profile of QT interval and QT interval variability in 172 healthy volunteers. *Pacing Clin. Electrophysiol.* 26 377–382. 10.1046/j.1460-9592.2003.00053.x 12687849

[B15] Bootsma M. Swenne C. A. Van Bolhuis H. H. Chang P. C. Cats V. M. Bruschke A. V. (1994). Heart rate and heart rate variability as indexes of sympathovagal balance. *Am. J. Physiol.* 266(4 Pt 2), H1565–H1571. 8184935 10.1152/ajpheart.1994.266.4.H1565

[B16] Brandt M. C. Priebe L. Böhle T. Südkamp M. Beuckelmann D. J. (2000). The ultrarapid and the transient outward K(+) current in human atrial fibrillation. their possible role in postoperative atrial fibrillation. *J. Mol. Cell Cardiol.* 32 1885–1896. 10.1006/jmcc.2000.1221 11013132

[B17] Bray M. S. Young M. E. (2008). Diurnal variations in myocardial metabolism. *Cardiovasc. Res.* 79 228–237. 10.1093/cvr/cvn054 18304930

[B18] Browne K. F. Prystowsky E. Heger J. J. Chilson D. A. Zipes D. P. (1983). Prolongation of the Q-T interval in man during sleep. *Am. J. Cardiol.* 52 55–59. 10.1016/0002-9149(83)90068-1 6858927

[B19] Bryant S. M. Wan X. Shipsey S. J. Hart G. (1998). Regional differences in the delayed rectifier current (IKr and IKs) contribute to the differences in action potential duration in basal left ventricular myocytes in guinea-pig. *Cardiovasc. Res.* 40 322–331. 10.1016/s0008-6363(98)00133-3 9893726

[B20] Buhr E. D. Takahashi J. S. (2013). Molecular components of the mammalian circadian clock. *Handb. Exp. Pharmacol.* 217 3–27. 10.1007/978-3-642-25950-0_1 PMC3762864 23604473

[B21] Buttgereit F. Doering G. Schaeffler A. Witte S. Sierakowski S. Gromnica-Ihle E. (2008). Efficacy of modified-release versus standard prednisone to reduce duration of morning stiffness of the joints in rheumatoid arthritis (CAPRA-1): a double-blind, randomised controlled trial. *Lancet* 371 205–214. 10.1016/s0140-6736(08)60132-4 18207016

[B22] Caballero R. de la Fuente M. G. Gómez R. Barana A. Amorós I. Dolz-Gaitón P. (2010). In humans, chronic atrial fibrillation decreases the transient outward current and ultrarapid component of the delayed rectifier current differentially on each atria and increases the slow component of the delayed rectifier current in both. *J. Am. Coll. Cardiol.* 55 2346–2354. 10.1016/j.jacc.2010.02.028 20488306

[B23] Carmeliet E. (2006). Repolarization reserve in cardiac cells. *J. Med. Biol. Eng.* 26 97–105.

[B24] Chen P. S. Chen L. S. Fishbein M. C. Lin S. F. Nattel S. (2014). Role of the autonomic nervous system in atrial fibrillation: pathophysiology and therapy. *Circ. Res.* 114 1500–1515. 10.1161/circresaha.114.303772 24763467 PMC4043633

[B25] Chen Y. Zhu D. Yuan J. Han Z. Wang Y. Qian Z. (2016). CLOCK-BMAL1 regulate the cardiac L-type calcium channel subunit CACNA1C through PI3K-Akt signaling pathway. *Can. J. Physiol. Pharmacol.* 94 1023–1032. 10.1139/cjpp-2015-0398 27376484

[B26] Christ T. Boknik P. Wöhrl S. Wettwer E. Graf E. M. Bosch R. F. (2004). L-type Ca2+ current downregulation in chronic human atrial fibrillation is associated with increased activity of protein phosphatases. *Circulation* 110 2651–2657. 10.1161/01.cir.0000145659.80212.6a 15492323

[B27] Cugini P. Di Palma L. Di Simone S. Lucia P. Battisti P. Coppola A. (1993). Circadian rhythm of cardiac output, peripheral vascular resistance, and related variables by a beat-to-beat monitoring. *Chronobiol. Int.* 10 73–78. 10.3109/07420529309064484 8443846

[B28] Curran M. E. Splawski I. Timothy K. W. Vincent G. M. Green E. D. Keating M. T. (1995). A molecular basis for cardiac arrhythmia: HERG mutations cause long QT syndrome. *Cell* 80 795–803. 10.1016/0092-8674(95)90358-5 7889573

[B29] Damiola F. Le Minh N. Preitner N. Kornmann B. Fleury-Olela F. Schibler U. (2000). Restricted feeding uncouples circadian oscillators in peripheral tissues from the central pacemaker in the suprachiasmatic nucleus. *Genes Dev.* 14 2950–2961. 10.1101/gad.183500 11114885 PMC317100

[B30] Degaute J. P. van de Borne P. Linkowski P. Van Cauter E. (1991). Quantitative analysis of the 24-hour blood pressure and heart rate patterns in young men. *Hypertension* 18 199–210. 10.1161/01.hyp.18.2.199 1885228

[B31] DiFrancesco J. C. DiFrancesco D. (2015). Dysfunctional HCN ion channels in neurological diseases. *Front. Cell Neurosci.* 6:174. 10.3389/fncel.2015.00071 PMC4354400 25805968

[B32] Dobrev D. Friedrich A. Voigt N. Jost N. Wettwer E. Christ T. (2005). The G protein-gated potassium current I(K,ACh) is constitutively active in patients with chronic atrial fibrillation. *Circulation* 112 3697–3706. 10.1161/circulationaha.105.575332 16330682

[B33] Du Pre B. C. Van Laake L. W. Meine M. Van der Heijden J. F. Doevendans P. A. Vos M. A. (2017). Analysis of 24-h rhythm in ventricular repolarization identifies QT diurnality as a novel clinical parameter associated with previous ventricular arrhythmias in heart failure patients. *Front Physiol.* 8:590. 10.3389/fphys.2017.00590 PMC5559512 28861002

[B34] Durgan D. J. Hotze M. A. Tomlin T. M. Egbejimi O. Graveleau C. Abel E. D. (2005). The intrinsic circadian clock within the cardiomyocyte. *Am. J. Physiol. Heart Circ. Physiol.* 289 H1530–H1541. 15937094 10.1152/ajpheart.00406.2005

[B35] Eckel-Mahan K. L. Patel V. R. de Mateo S. Orozco-Solis R. Ceglia N. J. Sahar S. (2013). Reprogramming of the circadian clock by nutritional challenge. *Cell* 155 1464–1478. 10.1016/j.cell.2013.11.034 24360271 PMC4573395

[B36] El-Sherif N. Turitto G. (2003). Torsade de pointes. *Curr. Opin. Cardiol.* 18 6–13. 12496496 10.1097/00001573-200301000-00002

[B37] Englund A. Behrens S. Wegscheider K. Rowland E. (1999). Circadian variation of malignant ventricular arrhythmias in patients with ischemic and nonischemic heart disease after cardioverter defibrillator implantation. European 7219 jewel investigators. *J. Am. Coll. Cardiol.* 34 1560–1568. 10.1016/s0735-1097(99)00369-1 10551707

[B38] Felici M. G. Spallone V. Maiello M. R. Gatta R. Civetta E. Frontoni S. (1991). Twenty-four hours blood pressure and heart rate profiles in diabetics with and without autonomic neuropathy. *Funct. Neurol.* 6 299–304. 1743546

[B39] Fletcher E. K. Kanki M. Morgan J. Ray D. W. Delbridge L. Fuller P. J. (2019). Cardiomyocyte transcription is controlled by combined MR and circadian clock signalling. *J. Endocrinol.* 10.1530/JOE-18-0584 Online ahead of print 30689544

[B40] Furlan R. Guzzetti S. Crivellaro W. Dassi S. Tinelli M. Baselli G. (1990). Continuous 24-hour assessment of the neural regulation of systemic arterial pressure and RR variabilities in ambulant subjects. *Circulation* 81 537–547. 10.1161/01.cir.81.2.537 2297860

[B41] Gardner R. T. Ripplinger C. M. Myles R. C. Habecker B. A. (2016). Molecular mechanisms of sympathetic remodeling and arrhythmias. *Circ. Arrhythm. Electrophysiol.* 9:e001359. 10.1161/CIRCEP.115.001359 PMC4730917 26810594

[B42] Gekakis N. Staknis D. Nguyen H. B. Davis F. C. Wilsbacher L. D. King D. P. (1998). Role of the CLOCK protein in the mammalian circadian mechanism. *Science* 280 1564–1569. 10.1126/science.280.5369.1564 9616112

[B43] Giacchetti S. Bjarnason G. Garufi C. Genet D. Iacobelli S. Tampellini M. (2006). Phase III trial comparing 4-day chronomodulated therapy versus 2-day conventional delivery of fluorouracil, leucovorin, and oxaliplatin as first-line chemotherapy of metastatic colorectal cancer: the European organisation for research and treatment of cancer chronotherapy group. *J. Clin. Oncol.* 24 3562–3569. 10.1200/jco.2006.06.1440 16877722

[B44] Gill S. Panda S. (2015). A smartphone app reveals erratic diurnal eating patterns in humans that can be modulated for health benefits. *Cell Metab.* 22 789–798. 10.1016/j.cmet.2015.09.005 26411343 PMC4635036

[B45] Gill S. Le H. D. Melkani G. C. Panda S. (2015). Time-restricted feeding attenuates age-related cardiac decline in Drosophila. *Science* 347 1265–1269. 10.1126/science.1256682 25766238 PMC4578815

[B46] Gonzalez de la Fuente M. Barana A. Gómez R. Amorós I. Dolz-Gaitón P. Sacristán S. (2013). Chronic atrial fibrillation up-regulates beta1-Adrenoceptors affecting repolarizing currents and action potential duration. *Cardiovasc. Res.* 97 379–388. 10.1093/cvr/cvs313 23060133

[B47] Green C. B. (2018). Circadian posttranscriptional regulatory mechanisms in mammals. *Cold Spring Harb. Perspect. Biol.* 10:a030692. 10.1101/cshperspect.a030692 28778869 PMC5983185

[B48] Guillaumond F. Dardente H. Giguère V. Cermakian N. (2005). Differential control of bmal1 circadian transcription by REV-ERB and ROR nuclear receptors. *J. Biol. Rhythms* 20 391–403. 10.1177/0748730405277232 16267379

[B49] Hatori M. Vollmers C. Zarrinpar A. DiTacchio L. Bushong E. A. Gill S. (2012). Time-restricted feeding without reducing caloric intake prevents metabolic diseases in mice fed a high-fat diet. *Cell Metab.* 15 848–860. 10.1016/j.cmet.2012.04.019 22608008 PMC3491655

[B50] Havel P. J. Uriu-Hare J. Y. Liu T. Stanhope K. L. Stern J. S. Keen C. L. (1998). Marked and rapid decreases of circulating leptin in streptozotocin diabetic rats: reversal by insulin. *Am. J. Physiol.* 274 R1482–R1491. 9612417 10.1152/ajpregu.1998.274.5.R1482

[B51] Hjalmarson A. Gilpin E. A. Nicod P. Dittrich H. Henning H. Engler R. (1989). Differing circadian patterns of symptom onset in subgroups of patients with acute myocardial infarction. *Circulation* 80 267–275. 10.1161/01.cir.80.2.267 2568893

[B52] Imai Y. Abe K. Sasaki S. Minami N. Munakata M. Nihei M. (1989). Exogenous glucocorticoid eliminates or reverses circadian blood pressure variations. *J. Hypertens.* 7 113–120. 2926131

[B53] Jaisser F. Swynghedauw B. Delcayre C. (2011). The mineralocorticoid receptor in heart: different effects in different cells. *Hypertension* 57 679–680. 10.1161/hypertensionaha.110.164962 21321302

[B54] Javed S. Gupta D. Lip G. Y. H. (2020). Obesity and atrial fibrillation: making inroads through fat. *Eur. Heart J. Cardiovasc. Pharmacother.* 10.1093/ehjcvp/pvaa013 Online ahead of print 32096865

[B55] Jeyaraj D. Haldar S. M. Wan X. McCauley M. D. Ripperger J. A. Hu K. (2012). Circadian rhythms govern cardiac repolarization and arrhythmogenesis. *Nature* 483 96–99. 10.1038/nature10852 22367544 PMC3297978

[B56] Jones D. K. Liu F. Vaidyanathan R. Eckhardt L. L. Trudeau M. C. Robertson G. A. (2014). hERG 1b is critical for human cardiac repolarization. *Proc. Natl. Acad. Sci. U.S.A.* 111 18073–18077. 10.1073/pnas.1414945111 25453103 PMC4273358

[B57] Jones E. M. Roti Roti E. C. Wang J. Delfosse S. A. Robertson G. A. (2004). Cardiac IKr channels minimally comprise hERG 1a and 1b subunits. *J. Biol. Chem.* 279 44690–44694. 10.1074/jbc.m408344200 15304481

[B58] Kaneko H. Kaitsuka T. Tomizawa K. (2020). Response to stimulations inducing circadian rhythm in human induced pluripotent stem cells. *Cells* 9:620. 10.3390/cells9030620 32143467 PMC7140533

[B59] Khan I. A. (2002). Clinical and therapeutic aspects of congenital and acquired long QT syndrome. *Am. J. Med.* 112 58–66. 10.1016/s0002-9343(01)01011-7 11812408

[B60] Kim S. M. Huang Y. Qin Y. Mizel D. Schnermann J. Briggs J. P. (2008). Persistence of circadian variation in arterial blood pressure in beta1/beta2-adrenergic receptor-deficient mice. *Am. J. Physiol. Regul. Integr. Comp. Physiol.* 294 R1427–R1434. 18305025 10.1152/ajpregu.00074.2008 PMC2386676

[B61] Lai L. P. Su M. J. Lin J. L. Tsai C. H. Lin F. Y. Chen Y. S. (1999). Measurement of funny current (I(f)) channel mRNA in human atrial tissue: correlation with left atrial filling pressure and atrial fibrillation. *J. Cardiovasc. Electrophysiol.* 10 947–953. 10.1111/j.1540-8167.1999.tb01265.x 10413374

[B62] Levi F. Okyar A. (2011). Circadian clocks and drug delivery systems: impact and opportunities in chronotherapeutics. *Expert. Opin. Drug Deliv.* 8 1535–1541. 10.1517/17425247.2011.618184 22097903

[B63] Levi F. Le Louarn C. Reinberg A. (1985). Timing optimizes sustained-release indomethacin treatment of osteoarthritis. *Clin. Pharmacol. Ther.* 37 77–84. 10.1038/clpt.1985.15 3880688

[B64] London B. Trudeau M. C. Newton K. P. Beyer A. K. Copeland N. G. Gilbert D. J. (1997). Two isoforms of the mouse ether-a-go-go-related gene coassemble to form channels with properties similar to the rapidly activating component of the cardiac delayed rectifier K+ current. *Circ. Res.* 81 870–878. 10.1161/01.res.81.5.870 9351462

[B65] Makino M. Hayashi H. Takezawa H. Hirai M. Saito H. Ebihara S. (1997). Circadian rhythms of cardiovascular functions are modulated by the baroreflex and the autonomic nervous system in the rat. *Circulation* 96 1667–1674. 10.1161/01.cir.96.5.1667 9315563

[B66] Martinez-Mateu L. Saiz J. Aromolaran A. S. (2019). Differential modulation of IK and ICa, L channels in high-fat diet-induced obese Guinea Pig Atria. *Front. Physiol.* 10:1212. 10.3389/fphys.2019.01212 PMC6773813 31607952

[B67] Maury E. Ramsey K. M. Bass J. (2010). Circadian rhythms and metabolic syndrome: from experimental genetics to human disease. *Circ. Res.* 106 447–462. 10.1161/circresaha.109.208355 20167942 PMC2837358

[B68] Millar-Craig M. W. Bishop C. N. Raftery E. B. (1978). Circadian variation of blood-pressure. *Lancet* 1 795–797. 85815 10.1016/s0140-6736(78)92998-7

[B69] Nattel S. (2002). New ideas about atrial fibrillation 50 years on. *Nature* 415 219–226. 10.1038/415219a 11805846

[B70] Novella Romanelli M. Sartiani L. Masi A. Mannaioni G. Manetti D. Mugelli A. (2016). HCN channels modulators: the need for selectivity. *Curr. Top. Med. Chem.* 16 1764–1791. 10.2174/1568026616999160315130832 26975509 PMC5374843

[B71] Oakley R. H. Cidlowski J. A. (2015). Glucocorticoid signaling in the heart: a cardiomyocyte perspective. *J. Steroid Biochem. Mol. Biol.* 153 27–34. 10.1016/j.jsbmb.2015.03.009 25804222 PMC4568128

[B72] Oosting J. Struijker-Boudier H. A. Janssen B. J. (1997). Autonomic control of ultradian and circadian rhythms of blood pressure, heart rate, and baroreflex sensitivity in spontaneously hypertensive rats. *J. Hypertens.* 15 401–410. 10.1097/00004872-199715040-00011 9211175

[B73] Ortiz-Caro J. Gonzalez C. Jolin T. (1984). Diurnal variations of plasma growth hormone, thyrotropin, thyroxine, and triiodothyronine in streptozotocin-diabetic and food-restricted rats. *Endocrinology* 115 2227–2232. 10.1210/endo-115-6-2227 6238818

[B74] Panda S. (2016). Circadian physiology of metabolism. *Science* 354 1008–1015. 10.1126/science.aah4967 27885007 PMC7261592

[B75] Panda S. Antoch M. P. Miller B. H. Su A. I. Schook A. B. Straume M. (2002). Coordinated transcription of key pathways in the mouse by the circadian clock. *Cell* 109 307–320. 10.1016/s0092-8674(02)00722-5 12015981

[B76] Peters R. W. Muller J. E. Goldstein S. Byington R. Friedman L. M. (1989). Propranolol and the morning increase in the frequency of sudden cardiac death (BHAT Study). *Am. J. Cardiol.* 63 1518–1520. 10.1016/0002-9149(89)90019-2 2729140

[B77] Pickel L. Sung H. K. (2020). Feeding rhythms and the circadian regulation of metabolism. *Front. Nutr.* 7:39. 10.3389/fnut.2020.00039 PMC7182033 32363197

[B78] Puckerin A. Aromolaran K. A. Chang D. D. Zukin R. S. Colecraft H. M. Boutjdir M. (2016). hERG 1a LQT2 C-terminus truncation mutants display hERG 1b-dependent dominant negative mechanisms. *Heart Rhythm* 13 1121–1130. 10.1016/j.hrthm.2016.01.012 26775140

[B79] Robles M. S. Humphrey S. J. Mann M. (2017). Phosphorylation is a central mechanism for circadian control of metabolism and physiology. *Cell Metab.* 25 118–127. 10.1016/j.cmet.2016.10.004 27818261

[B80] Rodriguez-Colon S. He F. Bixler E. O. Fernandez-Mendoza J. Vgontzas A. N. Berg A. (2014). The circadian pattern of cardiac autonomic modulation and obesity in adolescents. *Clin. Auton. Res.* 24 265–273. 10.1007/s10286-014-0257-7 25358502 PMC4267540

[B81] Sale H. Wang J. O’Hara T. J. Tester D. J. Phartiyal P. He J. Q. (2008). Physiological properties of hERG 1a/1b heteromeric currents and a hERG 1b-specific mutation associated with long-QT syndrome. *Circ. Res.* 103:e81-95. 10.1161/CIRCRESAHA.108.185249 PMC2761010 18776039

[B82] Sanguinetti M. C. Jurkiewicz N. K. (1990). Two components of cardiac delayed rectifier K+ current. differential sensitivity to block by class III antiarrhythmic agents. *J. Gen. Physiol.* 96 195–215. 10.1085/jgp.96.1.195 2170562 PMC2228985

[B83] Schroder E. A. Burgess D. E. Zhang X. Lefta M. Smith J. L. Patwardhan A. (2015). The cardiomyocyte molecular clock regulates the circadian expression of Kcnh2 and contributes to ventricular repolarization. *Heart Rhythm* 12 1306–1314. 10.1016/j.hrthm.2015.02.019 25701773 PMC4541807

[B84] Schroder E. A. Lefta M. Zhang X. Bartos D. C. Feng H. Z. Zhao Y. (2013). The cardiomyocyte molecular clock, regulation of Scn5a, and arrhythmia susceptibility. *Am. J. Physiol. Cell Physiol.* 304 C954–C965. 23364267 10.1152/ajpcell.00383.2012 PMC3651636

[B85] Scott E. M. Carter A. M. Grant P. J. (2008). Association between polymorphisms in the Clock gene, obesity and the metabolic syndrome in man. *Int. J. Obes.* 32 658–662. 10.1038/sj.ijo.0803778 18071340

[B86] Shea S. A. Hilton M. F. Hu K. Scheer F. A. (2011). Existence of an endogenous circadian blood pressure rhythm in humans that peaks in the evening. *Circ. Res.* 108 980–984. 10.1161/circresaha.110.233668 21474818 PMC3086568

[B87] Shusterman V. Warman E. London B. Schwartzman D. (2012). Nocturnal peak in atrial tachyarrhythmia occurrence as a function of arrhythmia burden. *J. Cardiovasc. Electrophysiol.* 23 604–611. 10.1111/j.1540-8167.2011.02263.x 22429736 PMC4569016

[B88] Siegel D. Black D. M. Seeley D. G. Hulley S. B. (1992). Circadian variation in ventricular arrhythmias in hypertensive men. *Am. J. Cardiol.* 69 344–347. 10.1016/0002-9149(92)90231-m 1734646

[B89] Statius van Eps R. G. van den Meiracker A. H. Boomsma F. Man in ’t Veld A. J. Schalekamp M. A. (1993). Partial preservation of nocturnal fall in blood pressure in patients with catecholamine-producing tumours. *J. Hypertens. Suppl.* 11 S168–S169. 8158326

[B90] Stramba-Badiale M. Priori S. G. Napolitano C. Locati E. H. Viñolas X. Haverkamp W. (2000). Gene-specific differences in the circadian variation of ventricular repolarization in the long QT syndrome: a key to sudden death during sleep? *Ital. Heart J.* 1 323–328. 10832806

[B91] Swoap S. J. Li C. Wess J. Parsons A. D. Williams T. D. Overton J. M. (2008). Vagal tone dominates autonomic control of mouse heart rate at thermoneutrality. *Am. J. Physiol. Heart Circ. Physiol.* 294 H1581–H1588. 18245567 10.1152/ajpheart.01000.2007

[B92] Takigawa M. Kawamura M. Noda T. Yamada Y. Miyamoto K. Okamura H. (2012). Seasonal and circadian distributions of cardiac events in genotyped patients with congenital long QT syndrome. *Circ. J.* 76 2112–2118. 10.1253/circj.cj-12-0213 22785222

[B93] Tanaka K. Ashizawa N. Kawano H. Sato O. Seto S. Nishihara E. (2007). Aldosterone induces circadian gene expression of clock genes in H9c2 cardiomyoblasts. *Heart Vessels* 22 254–260. 10.1007/s00380-006-0968-3 17653520

[B94] Tong M. Watanabe E. Yamamoto N. Nagahata-Ishiguro M. Maemura K. Takeda N. (2013). Circadian expressions of cardiac ion channel genes in mouse might be associated with the central clock in the SCN but not the peripheral clock in the heart. *Biol. Rhythm Res.* 44 519–530. 10.1080/09291016.2012.704801 23420534 PMC3570950

[B95] Umemura Y. Maki I. Tsuchiya Y. Koike N. Yagita K. (2019). Human circadian molecular oscillation development using induced pluripotent stem cells. *J. Biol. Rhythms* 34 525–532. 10.1177/0748730419865436 31368392 PMC6732938

[B96] van den Berg M. P. Haaksma J. Veeger N. J. Wilde A. A. (2006). Diurnal variation of ventricular repolarization in a large family with LQT3-Brugada syndrome characterized by nocturnal sudden death. *Heart Rhythm* 3 290–295. 10.1016/j.hrthm.2005.11.023 16500301

[B97] van Mil A. Balk G. M. Neef K. Buikema J. W. Asselbergs F. W. Wu S. M. (2018). Modelling inherited cardiac disease using human induced pluripotent stem cell-derived cardiomyocytes: progress, pitfalls, and potential. *Cardiovasc. Res.* 114 1828–1842. 10.1093/cvr/cvy208 30169602 PMC6887927

[B98] Veerman D. P. Imholz B. P. Wieling W. Wesseling K. H. van Montfrans G. A. (1995). Circadian profile of systemic hemodynamics. *Hypertension* 26 55–59. 10.1161/01.hyp.26.1.55 7607733

[B99] Velasco A. Huerta I. Marin B. (1988). Plasma corticosterone, motor activity and metabolic circadian patterns in streptozotocin-induced diabetic rats. *Chronobiol. Int.* 5 127–135. 10.3109/07420528809079553 3401978

[B100] Vyas V. Lambiase P. (2019). Obesity and atrial fibrillation: epidemiology, pathophysiology and novel therapeutic opportunities. *Arrhythm. Electrophysiol. Rev.* 8 28–36. 10.15420/aer.2018.76.2 30918664 PMC6434511

[B101] Wang Y. Johnsen A. Olieslagers S. Cox C. Bucchi A. Wegner S. (2016). Circadian rhythm in heart rate is due to an intrinsic circadian clock in the sinus node. *Eur. Heart J.* 37:618.

[B102] Wang T. A. Yu Y. V. Govindaiah G. Ye X. Artinian L. Coleman T. P. (2012). Circadian rhythm of redox state regulates excitability in suprachiasmatic nucleus neurons. *Science* 337 839–842. 10.1126/science.1222826 22859819 PMC3490628

[B103] Woon P. Y. Kaisaki P. J. Bragança J. Bihoreau M. T. Levy J. C. Farrall M. (2007). Aryl hydrocarbon receptor nuclear translocator-like (BMAL1) is associated with susceptibility to hypertension and type 2 diabetes. *Proc. Natl. Acad. Sci. U.S.A.* 104 14412–14417. 10.1073/pnas.0703247104 17728404 PMC1958818

[B104] Xie Y. Tang Q. Chen G. Xie M. Yu S. Zhao J. (2019). New insights into the circadian rhythm and its related diseases. *Front. Physiol.* 10:682. 10.3389/fphys.2019.00682 PMC6603140 31293431

[B105] Yamashita T. Sekiguchi A. Iwasaki Y. K. Sagara K. Iinuma H. Hatano S. (2003). Circadian variation of cardiac K+ channel gene expression. *Circulation* 107 1917–1922. 10.1161/01.cir.0000058752.79734.f0 12668525

[B106] Young M. E. Razeghi P. Cedars A. M. Guthrie P. H. Taegtmeyer H. (2001). Intrinsic diurnal variations in cardiac metabolism and contractile function. *Circ. Res.* 89 1199–1208. 10.1161/hh2401.100741 11739286

[B107] Young M. E. Wilson C. R. Razeghi P. Guthrie P. H. Taegtmeyer H. (2002). Alterations of the circadian clock in the heart by streptozotocin-induced diabetes. *J. Mol. Cell Cardiol.* 34 223–231. 10.1006/jmcc.2001.1504 11851361

[B108] Zhang R. Lahens N. F. Ballance H. I. Hughes M. E. Hogenesch J. B. (2014). A circadian gene expression atlas in mammals: implications for biology and medicine. *Proc. Natl. Acad. Sci. U.S.A.* 111 16219–16224. 10.1073/pnas.1408886111 25349387 PMC4234565

